# Prostate tuberculosis mimicking prostate cancer: Case report and literature review

**DOI:** 10.1097/MD.0000000000036172

**Published:** 2023-11-24

**Authors:** Yu Li, Siyu Dan, Fang Yang, Xiuli He, Ling He, Wensheng Yue

**Affiliations:** a Department of Ultrasound, Affiliated Hospital of North Sichuan Medical College, Nanchong City, China; b Department of Ultrasound, Nanchong Central Hospital, The Second Clinical Medical College, North Sichuan Medical College (University), Nanchong, China; c Sichuan Key Laboratory of Medical Imaging, North Sichuan Medical College, Nanchong, China.

**Keywords:** CONTRAST-enhanced ultrasound, nuclear magnetic resonance imaging, prostate tuberculosis, shear wave elastography, sonographic characteristics

## Abstract

**Rationale:**

Prostate tuberculosis (PTB) has no specific symptoms, or insidious presentation in male reproductive system tuberculosis, and is difficult to detect in the early stage. When PTB develops to the late stage, it leads to disease progression and irreversible organ and tissue damage. At present, the imaging manifestations of prostate tuberculosis vary and are not well known to imaging physicians and urologists.

**Diagnoses and Interventions:**

This case was a PTB patient, whose main manifestation was elevated serum prostate-specific antigen and the diagnosis was confirmed by ultrasound-guided prostate biopsy. We analyzed the imaging performance of various imaging techniques, and summarized and explored the imaging characteristics reported in the previous literature, with the aim of improving the early detection rate and providing evidence-based practice for early regular antituberculosis treatment in PTB.

**Outcomes:**

The multiparametric transrectal ultrasound performance of PTB is characteristic, and can be used for the differential diagnosis of prostate cancer causing elevated prostate-specific antigen levels in aged men.

## 1. Introduction

Even to this day, tuberculosis remains a global public health problem that poses a serious public health risk, especially in developing countries. The urinary system is one of the common sites of involvement of extrapulmonary tuberculosis (TB) accounting for 30% to 40% of cases.^[1]^ Among them, prostatic tuberculosis (PTB) is mainly disseminated to the prostate caused by the spread of distant lesions via the hematogenous route. Meanwhile, it may also be caused by renal tuberculosis descending to the prostate. In the past, the diagnosis of PTB is made by histologic analysis after transurethral resection and less reported. Currently, the common imaging tools used to evaluate prostate disease are multi-parameter magnetic resonance imaging (mp-MRI) and transrectal ultrasound (TRUS). PTB is often asymptomatic in the early stage, and imaging features have not been summarized, its diagnosis has been extremely challenging.^[2]^ In April 2022, a patient with prostate tuberculosis was admitted to the Affiliated Hospital of North Sichuan Medical College with elevated serum prostate-specific antigen (PSA). The multiple imaging presentations are listed below, and the literature related to the imaging features of PTB is reviewed and summarized.

## 2. Case report

### 2.1. Basic information

A 52-year-old male patient had recurrent dysuria with nocturia for 1+ years, the main symptoms were laborious urination, thinning of urine line, difficulty or impassability of urination, and endless urination, frequent urination, urgent urination, etc., without chills and fever, nausea, and vomiting. He was diagnosed and treated with “prostatic hyperplasia” in other hospitals. However, the curative effect was poor. On April 20, 2022, he was admitted to residential treatment with elevated serum PSA (tPSA: 12.42 μg/L, fPSA: 1.27 μg/L, fPSA/tPSA: 0.102). After admission, he underwent a series of examinations, digital rectal examination showed a Grade II enlarged prostate with medium texture and smooth surface, no nodules were touched. A urine routine examination showed that urine leukocyte count of 29/μL and a red blood cell count of 26/μL. Transabdominal ultrasonography examination showed a cystic nodule with a thin and smooth wall and clear inside with a diameter of approximately 1.0 cm seen in both renal parenchymas respectively. The obvious abnormality was not found in the ureter and bladder. Computed X-ray tomography showed scattered small nodules in both lungs, with diameters of about 0.2 to 0.6 cm, and a few fibrotic lesions in the upper lobe and sublingual segment of the left lung were found. Meanwhile, HIV serological test was negative, and the laboratory data such as whole blood cell count, biochemical examination, and coagulation time were normal.

### 2.2. Imaging diagnosis

**Figure 1.**
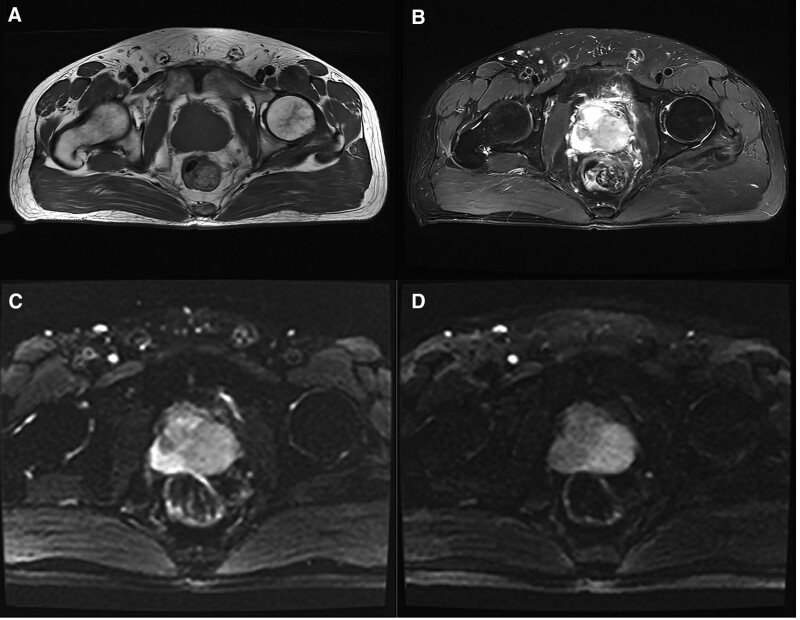
mp-MRI performance: (A) Equal signal displayed on T1WI transverse position; (B) Low signal on compression lipid T2WI; (C) Diffusion limitation on DWI and low signal on ADC; (D) Enhancement MRI showed that the left peripheral zone was a significantly inhomogeneous enhancement. ADC = apparent diffusion coefficient; mp-MRI = multi-parameter magnetic resonance imaging.

Contrast-enhanced magnetic resonance revealed an enlarged prostate with a mass-like abnormal signal shadow in the peripheral zone, an equal signal in T1WI, a slightly high signal in compression lipid T2WI, and a high signal in DWI. Furthermore, the lesion invaded the left seminal vesicle gland the left peripheral zone heterogeneous enhanced significantly, and pelvic lymph nodes showed. Finally, prostate cancer (PCa) was suspected (Fig. 1).

**Figure 2.**
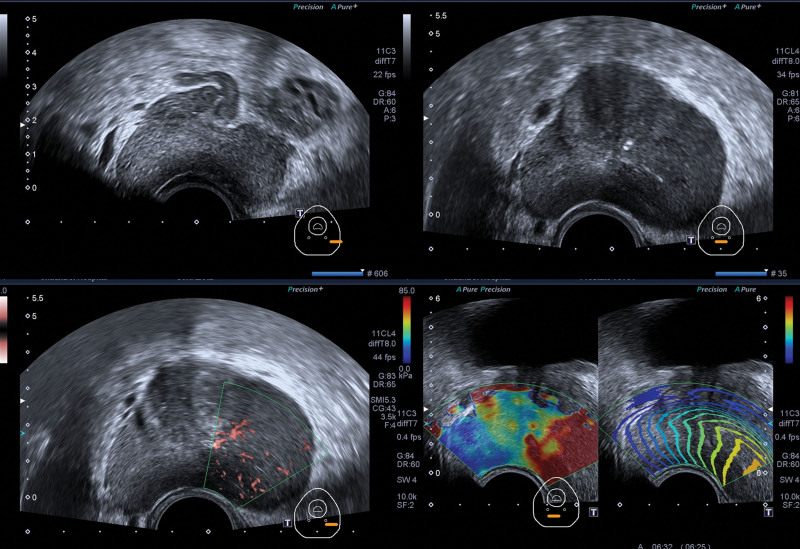
TRUS presentation: (A) The volume of the peripheral zone of the prostate was enlarged, the left peripheral zone was fuller than the right, and the gray-scale images were less uniform. (B) Color Doppler showed blood flow signal was of left peripheral zone Adler grade I. (C) Involvement of the left vesicular gland. (D) SWE showed the left peripheral zone was hard and displayed red, and the right peripheral zone was softer and displayed blue. TRUS = transrectal ultrasound.

**Figure 3.**
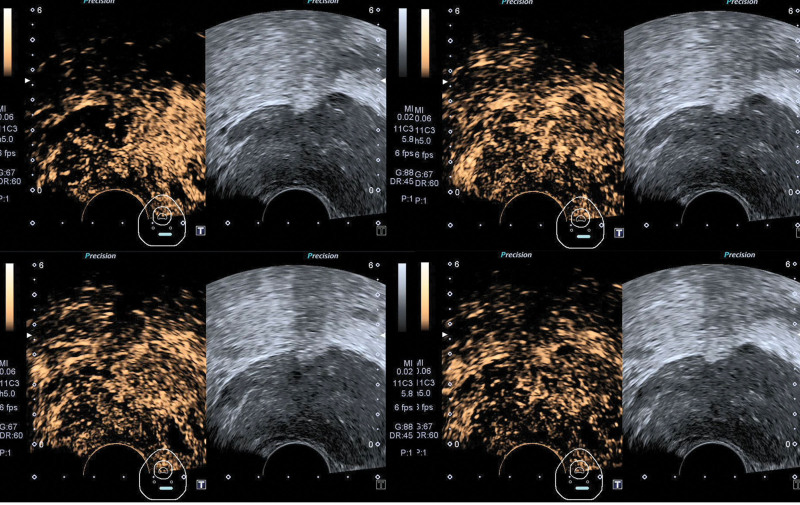
CETRUS presentation: (A) The left peripheral zone showed an inhomogeneous high enhancement pattern compared with the right side at the 18th. (B) The prostate is inhomogeneous after enhancement at the 29ths. (C and D) Ultrasound contrast agent in the whole prostate was fading simultaneously. (A)–(D) show the little region of the left peripheral zone (L1 and 2) without enhancement. CETRUS = contrast-enhanced transrectal ultrasound.

Conventional TRUS revealed that PTB had an enlarged peripheral zone of the prostate with a fuller morphology, especially on the left side. The echogenicity of the peripheral zone was inhomogeneous, but there was no obvious space-occupying effect. The real-time 2-dimensional shear wave elastography (SWE) was observed. In the stable state, the prostate images of the right peripheral zone were filled with a more homogeneous blue, and the elastic modulus of the region of interest was measured as *E*_mean_ = 19.7 kPa. Meanwhile, the left peripheral zone displayed red color, and an elastic abnormal area presented filling defects, the elastic modulus of the region of interest was measured as *E*_mean_ = 113.3 kPa (Fig. 2). The contrast-enhanced transrectal ultrasound (CEUS) examination: 2.4 mL of prepared SonoVue contrast agent was injected into the superficial vein of the elbow, followed by 5 mL of saline was rapidly pushed into the vein and the dynamic images from the start of the injection of contrast agent to 3 minutes after the injection were saved. In the following work, the perfusion patterns, contrast enhancement, and fading characteristics in various parts and abnormal areas of the prostate were observed. In this CEUS case, the left peripheral zone showed rapid and high enhancement compared with the right peripheral zone. Furthermore, the distribution of contrast medium was inhomogeneities, and a small non-enhanced area was seen in the enhanced prostate tissue. However, the right peripheral zone of the prostate displayed a low enhancement pattern, and the prostate tissue as a whole was contoured simultaneously (Fig. 3). According to the above findings, we considered mostly prostatitis.

### 2.3. Pathological result

**Figure 4.**
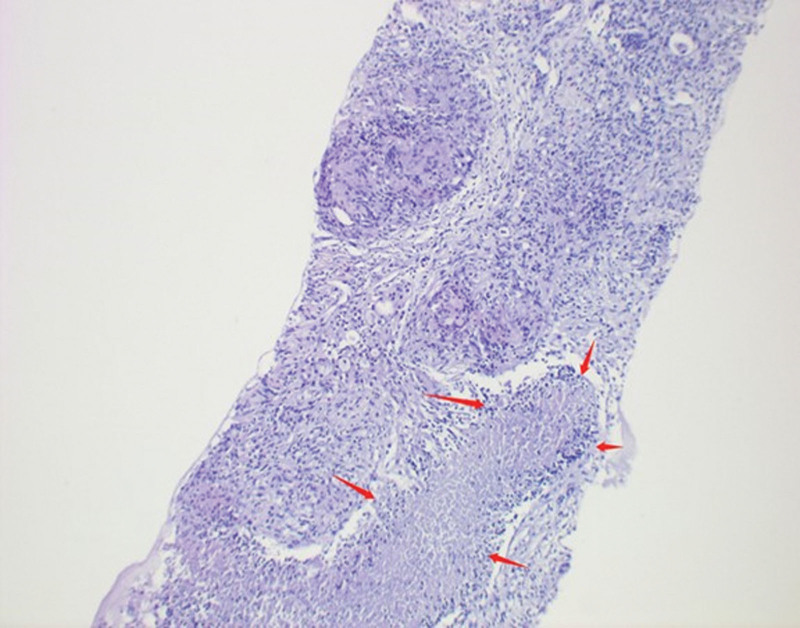
Pathological histogram: Red arrows pointed to areas of coagulative necrosis in the left peripheral zone (L1 and 2) (hematoxylin-eosin staining ×20).

According to the 2020, Chinese Society Of Clinical Oncology guidelines, when elevated PSA and abnormal MRI manifestations, prostate biopsy should be considered. In this case, was performed transperineal ultrasound-guided prostate 10 + X system biopsy and obtained 10 tissue strips. The pathological results found that both the left and right glands of the prostate were benign lesions, with scattered or focal lymphocytic infiltration in the interstitium, and granulomatous nodules with central coagulative necrosis were seen in the interstitium around the left peripheral zone and transition zone (Fig. 4).

### 2.4. Therapeutic intervention

In previous medical history, the patient had been vaccinated with the BCG TB vaccine in childhood and had no history of tuberculosis infection, and there was no chronic cough, long-term fever, weight loss, or tuberculosis infection in the patient’s family. However, in the laboratory examination, there was a weakly positive tuberculosis antibody (TBAB), and the urine Mycobacterium tuberculosis DNA test and interferon γ release test were positive. The patient was reviewed after 6 months of anti-tuberculosis treatment and the PSA level was reduced, and TRUS examination did not reveal any significant lesions.

## 3. Discussion

PTB is a form of idiopathic infective granulomatous prostatitis, which often occurs in seminal vesicle tuberculosis and epididymal tuberculosis simultaneously. PTB is usually asymptomatic or subclinical in the early stage and nonspecific irritating micturition in the late stage. PTB is a serious and insidious disease and is often found accidentally in partial prostatectomy or biopsy. In PTB examination, prostatic hypertrophy, palpation with or without pain, and hard areas can be found by digital rectal examination. Meanwhile, PSA may be normal or elevated, and urine analysis and urine culture are usually negative. However, PTB is difficult to distinguish from nonspecific prostatitis, benign prostatic hyperplasia, and PCa, because they have similar clinical and imaging changes, resulting in difficult and delayed diagnosis.^[3]^ Therefore, imaging manifestations of PTB should be more recognized, and early diagnosis should be improved. In PCa, because of the tight intercellular space connection and less water, the diffusion of water molecules is often limited or not diffused, and the apparent diffusion coefficient (ADC) signal is low.

We reviewed the literature on the imaging features of prostate tuberculosis reported, Most of them were observational studies or case reports with small sample sizes. The clinical data and imaging features (Table 1) of prostate tuberculosis-related cases in the previous literature are summarized in Table 2 and Table 3.

**Table 1:** TRUS features of 17 patients.

No.	References	Age	Volume	TRUS	CETRUS
1	^[12]^	23	Normal	Left PZ hypoechoic	Left PZ non-enhanced area
2	^[12]^	19	Normal	TZ low echo area	TZ non-enhanced area
3	^[12]^	24	Normal	Diffuse hypoechoic of prostate	A large area of prostate non-enhanced area
4	^[12]^	23	Enlarged	Multiple hypoechoic areas in PZ	PZ non-enhanced area
5	^[12]^	56	Enlarged	Large hypoechoic area in the prostate	A large area of prostate non-enhanced area
6	^[12]^	24	Normal	Diffuse hypoechoic peripheral gyrus	Virtually no enhancement of PZ
7	^[12]^	39	Normal	Diffuse hypoechoic of the prostate	PZ non-enhanced nodule
8	^[12]^	44	Normal	TZ with multiple hypoechoic	TZ scattered the non-enhanced area
9	^[12]^	17	Normal	Scattered hypoechoic areas of the prostate	Little to no enhancement of the entire prostate
10	^[12]^	31	Enlarged	Diffuse hypoechoic of the prostate	Large area of prostate non-enhanced area
11	^[12]^	36	Enlarged	Diffuse hypoechoic of PZ	Large area of prostate non-enhanced area
12	^[12]^	33	Normal	Hypoechoic area	PZ non-enhanced nodule
13	^[2]^	60	Enlarged	Multiple hypoechoic in prostate	NA
14	^[2]^	40	Enlarged	Hypoechoic area	NA
15	^[2]^	48	Enlarged	Hypoechoic area	NA
16	^[2]^	60	Enlarged	Multiple hypoechoic areas	NA
17	This case	58	Enlarged	Diffuse lesions of the PZ	Left PZ non-enhanced nodule

The volume of the prostate > 20 mL is enlarged, and ≤20 mL is normal.

CETRUS = contrast-enhanced transrectal ultrasound, PZ = peripheral zone, TRUS = transrectal ultrasound, TZ = transitional zone.

**Table 2:** The clinical manifestation of the 14 patients.

Clinical manifestation	Number of cases	Percentage (%)
Fever	2	14.3
Duration of symptoms	6	42.8
Pulmonary tuberculosis	4	28.6
Storage symptoms	14	100
Hematuria	5	35.7
Lumbar pain	4	28.6
Urine-WBC	6	42.8
Urine-RBC	4	28.6
PSA elevation	7	50

PSA = prostate-specific antigen.

**Table 3:** Multi-parametric MRI performance of 13 patients.

No.	Reference	Age	Lesion type	T1WI	T2WI	DWI	ADC	DCE
1	^[11]^	48	Nodular	Iso-signal	Low signal	Unrestricted dispersion	Low	Edge enhancement
2	^[11]^	63	Nodular	Iso-signal	Low signal	Unrestricted dispersion	Low	NA
3	^[11]^	64	Diffuse	Iso-signal	Low signal	Slightly limited dispersion	Low	NA
4	^[11]^	67	Diffuse	Iso-signal	Low signal	Slightly limited dispersion	Low	NA
5	^[11]^	62	Diffuse	Iso-signal	Low signal	Restricted diffusion	Low	NA
6	^[11]^	59	Diffuse	Iso-signal	Low signal	Restricted diffusion	Low	NA
7	^[10]^	59	NA	Na	Low signal	Restricted diffusion	Low	NA
8	^[10]^	65	NA	NA	Low signal	Restricted diffusion	Low	NA
9	^[6]^	64	Nodular	NA	Low signal	Restricted diffusion	Low	Ring Enhancement
10	^[6]^	57	Diffuse	NA	Low signal	Restricted diffusion	Low	NA
11	^[6]^	46	Diffuse	NA	Low signal	Restricted diffusion	Low	Significantly enhanced
12	^[6]^	65	Nodular	NA	Slightly higher signal	Restricted diffusion	Low	Low-level enhancement, edge enhancement
13	This case	58	Nodular	Iso-signal		Restricted diffusion	Low	Significantly enhanced

ADC = apparent diffusion coefficient; DCE = dynamic contrast-enhanced.

And as early as 1995, Tajima et al^[4]^ reported a case of MR presentation of prostate tuberculosis in an elderly man. The clinical data and imaging features of cases related to prostate tuberculosis in previous literature are summarized below.

Previous literature concluded that high-resolution ultrasound is the best method to evaluate epididymis, testis, scrotum, and vas deferens in male genital tuberculosis,^[5]^ while mp-MRI was determined to be the best method to evaluate the prostate, seminal vesicle, and ejaculatory duct.^[6]^ To review and summarize the mp-MRI imaging features of prostate tuberculosis in the previous literature. Suzuki et al^[7]^ classified PTB into 3 types: nodular type, diffuse type, and cystic mural nodule type, of which diffuse type was the most common. The mp-MRI showed iso-signal on T1WI, the diffuse or focal hypoechoic signal on T2WI, and presented mixed high-low signal due to different pathological stages of PTB. The nodular type of PTB had pleomorphic characteristics feature and clumps of abnormal signals could be seen in MRI. According to the Prostate Imaging Reporting and Data System version (PI-RADS) v2.1, the T2W score is the dominant factor that determines the PI-RADS assessment category in TB, and restricted diffusion is also a feature of malignant tumors. T2WI of PCa often shows low signal.^[8]^ The DWI is a signal reflecting the diffusion ability of water molecules, which PCa often presents with significantly limited or no diffusion and low ADC value due to tight intercellular connections and less water. DWI of PTB can also show limited diffusion or limited, low ADC value, but the signal is higher than that of PCa, which has differential significance with PCa.^[9]^ Dynamic contrast-enhanced magnetic resonance imaging (DCE-MRI) showed that most PCa is an early enhancement in DCE. Dynamic enhanced magnetic resonance imaging (DCE-MRI), most PCas show early enhancement in DCE, and a fast-rising and then declining outflow type curve, the DCE-MRI performance of prostate tuberculosis has not been reported systematically.^[10]^ In this case, DCE-MRI appeared to have a significant enhancement in the prostate, which needs further study. Some MRS features have been reported to differentiate PTB from cancer.^[11]^ PTB is a benign disease that will not significantly accelerate cell proliferation or eliminate citric acid metabolism. Therefore, the citric acid level should be within the normal range, which is also a distinguishing point to differentiate from PCa. MRI is helpful to determine the location and extent of the lesion, and functional imaging can provide pathological, biochemical and metabolic information of the prostate, which is useful for biopsy location, diagnosis, treatment guidance and prognosis judgment.

Reviewing the previous literature, the TRUS imaging of PTB has no obvious and specific features, there may be prostate enlargement, hypoechoic lesions, and calcification caused by caseous necrosis. TRUS of nodular PTB mostly shows hypoechoic lesions, similar to PCa with poor Gleason score or focal PCa, which are difficult to distinguish by conventional ultrasound. In this case, the TRUS examination of PTB showed that the prostatic capsule was smooth, and the volume of the peripheral zone was increased with hypoechoic and diffuse changes, without obvious local space-occupying effect. It is different from PCa diffuse lesions, such as incomplete prostatic capsule, parenchymal nodular heterogeneity, and partial extraprostatic invasion. In this case, SWE was used to evaluate the hardness changes in local PTB lesions, and no relevant report was found. The pathological results of the right peripheral zone of the prostate demonstrated that the prostate was mildly inflammatory, with SWE displaying a blue color consistent with that of normal prostate tissue, while the left peripheral zone showed hard tissue with abnormal elasticity, which displayed in the red or color filling defect. The imaging pattern and measurement values partially overlap with those of PCa, and further study with a larger sample size is needed.

The pathological findings of the right peripheral zone suggest a milder degree of inflammatory lesions, with SWE showing a more consistent blue color with normal prostate tissue, while the left peripheral zone shows a harder red color and abnormal elasticity with elastic filling defects. The imaging pattern and measurement values partially overlap with those of PCa, and further study with a larger sample size is needed. The contrast-enhanced ultrasound patterns of PTB varied due to the pathological structure and the stage of disease progression. In this case, the imaging pattern of dynamic ultrasonography (CEUS) was consistent with the pathological findings of the puncture, with the right lobe of the prostate showing hyperenhancement. Yang Gao et al^[12]^ reported that the histopathological examination of the low enhancement area mainly included inflammatory cell infiltration, vasodilatation, and small perivascular tuberculous granulomas, suggesting an early stage of cicatricial inflammation. The pathological results of the high enhancement area in the left peripheral zone confirmed granulomatous inflammation, while the non-enhancement area may be related to tuberculous granuloma, caseous necrosis, and incomplete destruction of glands. Previous literature^[13]^ reported that about 83.3% of PTB CE-TRUS had non-enhanced areas, which were also proved to be caseous necrosis or purulent cavity by pathology. However, CEUS showed no enhancement area in PCa with liquefaction necrosis, but the liquefaction range was usually small. If CETRUS appeared in a large non-perfusion area, the possibility of tuberculosis increased. Meanwhile, TRUS shows that PTB caseous necrosis is consistent with the echo of surrounding glands, which is difficult to identify. CEUS can help to determine the location and area of necrosis, assist with prostate biopsy, reduce the number of puncture needles, and improve the detection rate. Therefore, various modes of ultrasound examination have certain feature characteristics in the diagnosis of PTB, which is suitable for screening and differential diagnosis of prostate diseases, especially ultrasound-guided puncture biopsy, and has high clinical application value.

## 4. Perspective

In conclusions, when meeting middle-aged and elderly men with urine storage symptoms, the possibility of prostate tuberculosis should be considered and inquired as the tuberculosis-related history and distinguish imaging features to avoid delay in diagnosis. Medical imaging examination can locate and measure the extent of the disease, distinguish the severity of the disease, guide prostate puncture, and evaluate the therapeutic effect.

## Acknowledgments

Thanks to my mentor Prof Yue Wensheng for teaching me the spark of thinking. Thanks to all those who participated in this study (including the patients).

## Author contributions

**Data curation:** Xiuli He.

**Formal analysis:** Ling He.

**Investigation:** Xiuli He.

**Methodology:** Siyu Dan.

**Resources:** Siyu Dan.

**Software:** Xiuli He.

**Supervision:** Wensheng Yue, Fang Yang.

**Validation:** Wensheng Yue, Ling He.

**Writing – original draft:** Yu Li.

**Writing – review & editing:** Yu Li, Fang Yang.
